# Supplementary material for: Citizen science data on urban forageable plants: a case study in Brazil
Source: GigaByte. 2024 Feb 21;2024:gigabyte107. doi: 10.46471/gigabyte.107 (PMC10905257; doi:10.46471/gigabyte.107)
Supplement: Translation

# Translation: Dados de Ciência Cidadã sobre Plantas Urbanas Forrageáveis: Um Estudo de Caso no Brasil

**Authors:** Filipi Miranda Soares, Luís Ferreira Pires, Maria Carolina Garcia, Lidio Coradin, Natalia Pirani Ghilardi-Lopes, Rubens Rangel Silva, Aline Martins de Carvalho, Anand Gavai, Yamine Bouzembrak, Benildes Coura Moreira dos Santos Maculan, Sheina Koffler, Uiara Bandineli Montedo, Debora Pignatari Drucker, Raquel Santiago, Maria Clara Peres de Carvalho, Ana Carolina da Silva Lima, Hillary Dandara Elias Gabriel, Stephanie Gabriele Mendonça de França, Karoline Reis de Almeida, Bárbara Junqueira dos Santos, Antonio Mauro Saraiva

**Affiliations:** ^1^ Escola Politécnica, Universidade de São Paulo, São Paulo, SP, 05508-010, Brazil; ^2^ Faculty of Electrical Engineering, Mathematics and Computer Science, University of Twente, Enschede, 7522 NB, Netherlands; ^3^ Programa de Pós Graduação em Arquitetura, Urbanismo e Design, Centro Universitário Belas Artes de São Paulo, São Paulo, SP, 04018-010, Brazil; ^4^ Plants for the Future Project, Brasília, DF, 70772-090, Brazil; ^5^ Centro de Ciências Naturais e Humanas, Universidade Federal do ABC, São Bernardo do Campo, SP, 09606-045, Brazil; ^6^ Centro Universitário Una, Belo Horizonte, MG, 30160-011, Brazil; ^7^ Departamento de Nutrição, Faculdade de Saúde Pública, Universidade de São Paulo, São Paulo, SP, 01246-904, Brazil; ^8^ Faculty of Behavioural, Management and Social Sciences (BMS), Industrial Engineering & Business Information Systems (IEBIS), University of Twente, Enschede, 7522 NB, Netherlands; ^9^ Information Technology Group, Wageningen University and Research, Wageningen, 6700 HB, Netherlands; ^10^ Programa de Pós-Graduação em Gestão & Organização do Conhecimento, Universidade Federal de Minas Gerais, Belo Horizonte, MG, 31270-901, Brazil; ^11^ Escola de Ciência da Informação, Universidade Federal de Minas Gerais, Belo Horizonte, MG, 31270-901, Brazil; ^12^ Instituto de Estudos Avançados, Universidade de São Paulo, São Paulo, SP, 05508-060, Brazil; ^13^ Embrapa Agricultura Digital, Campinas, SP, 13083-886, Brazil; ^14^ Faculdade de Nutrição, Universidade Federal de Goiás, Goiânia, GO, 74605-080, Brazil; ^15^ Escola de Artes, Ciências e Humanidades, Universidade de São Paulo, São Paulo, SP, 03828-000, Brazil; ^16^ Instituto de Pesquisas Energéticas e Nucleares, Universidade de São Paulo, São Paulo, SP, 05508-000, Brazil

## Abstract

Este artigo apresenta dois bancos de dados derivados do projeto Pomar Urbano. O primeiro banco de dados é um catálogo abrangente de espécies de plantas frutíferas para uso humano, nativas ou introduzidas no Brasil. O segundo banco de dados, obtido a partir da plataforma iNaturalist, documenta a distribuição e o monitoramento dessas plantas em áreas urbanas por todo o Brasil. O estudo abrange dados de todas as capitais das 27 unidades federativas brasileiras, focando nas dez cidades que mais contribuíram com observações até agosto de 2023. A pesquisa enfatiza a importância da ciência cidadã no monitoramento da biodiversidade urbana e seu potencial para contribuir em diversos campos, incluindo alimentação e nutrição, indústria criativa, estudo da fenologia das plantas e aplicações de aprendizado de máquina. Espera-se que estes bancos de dados sirvam como recurso para futuros estudos em forrageamento urbano, segurança alimentar, serviços ecossistêmicos culturais e sustentabilidade ambiental.

## Descrição dos dados

Este artigo apresenta dois bancos de dados relacionados a plantas frutíferas brasileiras. O Banco de Dados 1 apresenta uma lista taxonômica dessas espécies de plantas, tanto nativas quanto introduzidas, que inclui nomes científicos abrangentes de plantas com frutos comestíveis para humanos. Este banco de dados contém nomes científicos (em níveis de espécie e família), nomes vernaculares em português, meios de estabelecimento (nativo ou introduzido), distribuição geográfica das espécies no Brasil e a última atualização de um determinado nome de espécie dentro do banco de dados. Uma documentação mais detalhada do banco de dados está disponível no Zenodo (veja a seção [1.]).

O Banco de Dados 2, hospedado no iNaturalist, compila observações das espécies listadas no Banco de Dados 1 de todas as capitais das 27 unidades federativas do Brasil. Detalhes sobre como acessar esses bancos de dados podem ser encontrados na seção [2.]. Obtido do iNaturalist em 22 de agosto de 2023, o banco de dados apresenta 47 colunas que capturam o tempo da observação, localização, licença e detalhes de identificação taxonômica. Ele fornece uma ampla decomposição taxonômica, cobrindo classificações desde reino até espécie, subespécie e variedade em alguns casos. Notavelmente, o iNaturalist permite a exportação de bancos de dados anotados com metadados Darwin Core [3.], garantindo a interoperabilidade de dados com inúmeras aplicações que usam este padrão, como o Global Biodiversity Information Facility (GBIF).

A interoperabilidade com o GBIF é vital por várias razões. Ela melhora a visibilidade e a acessibilidade dos dados de biodiversidade em todo o mundo, facilitando para pesquisadores e formuladores de políticas o acesso a essas informações [4.]. O GBIF é considerado “o recurso mais extenso que fornece acesso a dados abertos e integrados sobre a ocorrência de espécies” [5., p. 1]. Isso é especialmente importante para apoiar a colaboração global em conservação e pesquisa sobre biodiversidade [4.–6.]. O GBIF também garante que o relatório de dados seja padronizado e de alta qualidade, crucial para estudos sobre biodiversidade [4., 5.]. Por fim, permite uma comparação e referência cruzada mais eficazes dos dados de biodiversidade de diferentes regiões e espécies, enriquecendo a pesquisa sobre a distribuição de espécies [5.].

## Referencial Teórico

### Sistemas alimentares urbanos

De acordo com o Programa das Nações Unidas para o Meio Ambiente [7.], o Brasil possui a biodiversidade mais rica do mundo, representando 15 a 20% da biodiversidade global [7., 8.]. Essa vasta biodiversidade se traduz em uma diversidade de alimentos disponíveis para os brasileiros. No entanto, como destaca Gomes [9.], essa rica biodiversidade não é adequadamente refletida nas dietas de vários grupos sociais no país. Na verdade, apenas 1,3% dos brasileiros consomem ativamente alimentos biodiversos. Localização geográfica, etnia, idade, insegurança alimentar, gênero e nível educacional influenciam esses padrões de consumo [9.].

A combinação única do clima tropical do Brasil e seus diversos ecossistemas cria condições favoráveis para o cultivo de uma grande variedade de frutas, muitas das quais são espécies nativas. Essas frutas não são cultivadas apenas em ambientes agrícolas, mas também são amplamente utilizadas na arborização urbana e contribuem para a estética de parques, praças e calçadas em várias cidades.

Plantas frutíferas que integram as florestas alimentares urbanas podem oferecer múltiplos benefícios, como aumentar a disponibilidade de alimentos, especialmente em áreas com acesso limitado a frutas frescas, abordando assim questões de escassez alimentar e melhorando a segurança nutricional [10.–12.]. Além disso, elas promovem a saúde e o bem-estar humano ao fomentar a conexão com a natureza, proporcionar oportunidades para atividade física e melhorar a qualidade do ar por meio do sequestro de carbono e mitigação da poluição [13.].

As florestas alimentares urbanas também contribuem para a preservação e promoção da biodiversidade, garantindo a conservação de variedades nativas de frutas e apoiando ecossistemas locais, fornecendo abrigo, alimento, locais para nidificação e melhorando as condições ambientais [11., 14., 15.].

Como explorado por Hurley *et al.* [16.], esses espaços verdes contribuem significativamente para outras aplicações não relacionadas à alimentação. Hurley e Emery [17.] investigam a relação intrincada entre as florestas urbanas e sua utilidade para alimentação, medicina, artesanato e outros fins, focando em espécies lenhosas. Eles destacam as conexões materiais desses espaços verdes e seus diversos usos em contextos artísticos e culturais, ressaltando sua importância multifacetada em ambientes urbanos [17.].

Ao reconhecer o potencial das infraestruturas verdes comestíveis, as áreas urbanas podem mitigar os impactos adversos da urbanização, ao mesmo tempo em que aprimoram a sustentabilidade e resiliência das cidades [11., 12.].

### Uso da ciência cidadã para o monitoramento da biodiversidade urbana

A ciência cidadã (CS) é um esforço de colaboração entre cientistas e o público, envolvendo a participação ativa de membros da sociedade na pesquisa científica. A participação em uma iniciativa de CS para coletar dados sobre plantas frutíferas localmente pode ser motivada por vários motivos, incluindo a curiosidade e o desejo pessoal pela iniciativa.

As plataformas de CS oferecem um ambiente propício para que os cientistas-cidadãos contribuam com a ciência e aprendam a importância de iniciativas dessa natureza. O iNaturalist, por exemplo, funciona como uma rede social on-line, que apoia uma comunidade de usuários que trocam informações sobre biodiversidade, promovendo o aprendizado colaborativo sobre o mundo natural [18.]. Ele oferece uma plataforma móvel, baseada na Web, que permite que os usuários façam *upload* de fotos para serem analisadas com Inteligência Artificial (IA), que vai indicar a identificação do organismo com base nessas fotos e em outras variáveis, como o local da observação.

A riqueza dos dados disponíveis na iNaturalist pode ser aproveitada para muitas iniciativas de pesquisa que se concentram no monitoramento da biodiversidade urbana. Alguns estudos utilizaram a iNaturalist para rastrear e compreender a presença de espécies exóticas. Por exemplo, na região de Tyumen na Rússia, os pesquisadores [19.] compararam os dados de toda a região com a área urbana de Tyumen para avaliar a distribuição de quatro espécies de plantas exóticas. Além disso, na cidade de Kiev, a expansão da lesma invasora dos Bálcãs (*Tandonia kusceri*) em paisagens urbanas foi estudada [20.].

Os insetos (classe *Insecta*) também têm sido um ponto focal de pesquisa em ambientes urbanos usando o iNaturalist. Os estudos exploraram vários aspectos da biodiversidade de insetos, como a riqueza de espécies de borboletas (*Lepidoptera: Rhopalocera*) em Los Angeles  [21.], a compilação de uma lista de verificação de espécies de hawkmoths (*Lepidoptera*) na cidade de Baguio, Filipinas [22.], e o monitoramento de abelhas carpinteiras orientais (*Xylocopa virginica*) em áreas urbanas dos Estados Unidos e do Canadá, tendo em vista detectar a perda de pigmento nas asas [23.]. Os projetos de CS que usam o iNaturalist também têm como alvo outros grupos de animais, incluindo estudos de mamíferos, aves, anfíbios e répteis, por exemplo, monitorando a presença de grandes mamíferos em centros urbanos na América do Norte durante a pandemia de COVID-19 [24.]. As raposas vermelhas urbanas e os coiotes foram monitorados usando observações do iNaturalist na América do Norte [25., 26.], assim como foram documentadas observações de pássaros na cidade de Chicago, Illinois, por meio do iNaturalist [27.]. Além disso, a pesquisa caracterizou a riqueza de espécies de anfíbios e répteis em parques ecológicos recreativos em Mérida, Yucatán, México [28.], e no Edith L. Moore Nature Sanctuary, localizado em Houston, Texas [29.].

Vários estudos com objetivos mais amplos também utilizaram dados do iNaturalist para entender interações ecológicas em cidades [30.–32.], monitorar a biodiversidade urbana [33., 34.], explorar o uso humano de áreas verdes urbanas na Europa [35.] e examinar práticas de manejo de insetos para agricultura urbana [36.].

Esta gama de estudos demonstra a versatilidade e o potencial do iNaturalist como ferramenta para explorar a biodiversidade urbana e as dinâmicas ecológicas. Também indica o potencial da plataforma na criação de bancos de dados de código aberto sobre plantas forrageáveis, conforme explorado por [37.].

## Métodos

### Banco de dados 1

A versão 3 da lista de espécies de plantas frutíferas nativas e exóticas cultivadas no Brasil foi construída com base em duas principais fontes, conforme recomendações de especialistas: o livro *Frutas Brasileiras e Exóticas Cultivadas* [38.] e a série de livros *Plantas para o Futuro*. Esta última inclui volumes especificamente dedicados a espécies de valor econômico de quatro regiões do Brasil, a saber, Sul [39.], Centro-Oeste [40.], Nordeste [41.] e Norte [8.]. Um botânico revisou cuidadosamente esta lista nas seguintes etapas:

(i) Verificação do Nome Científico: A principal tarefa envolveu a verificação de cada nome científico para assegurar a existência, correção e validade do nome da espécie. A revisão focou em determinar se os nomes das espécies eram aceitos ou sinônimos no momento atual. Recursos-chave para essa verificação incluíram o catálogo Flora e Funga do Brasil [42.], Tropicos.org do Jardim Botânico do Missouri [43.], o Índice Internacional de Nomes de Plantas [44.], Kew Botanic Gardens Plants of the World Online [45.], e o Catálogo de Tipos de Plantas Vasculares do Jardim Botânico de Nova York [46.]. É importante notar que os nomes científicos são dinâmicos; uma espécie considerada válida hoje pode se tornar um sinônimo amanhã, e vice-versa. Portanto, a atualização desta lista será contínua.
(ii) Verificação de Origem: classificação da espécie como nativa ou introduzida. Isso envolveu extensa pesquisa online e consulta à literatura relevante, com foco principalmente na série de livros *Plantas para o Futuro* [8., 39.–41.].
(iii) Duas novas colunas foram adicionadas à Versão 3 da lista: Família Botânica e Distribuição Geográfica. As informações para essas colunas foram obtidas principalmente do catálogo Flora e Funga do Brasil e Tropicos.org.
(iv) Tratamento de Sinônimos: muitas espécies incluídas na Versão 2 da lista, a partir dos livros de referência, foram identificadas como sinônimos de acordo com os catálogos online consultados. Nestes casos, os nomes científicos válidos atuais substituíram os sinônimos.
(v) Espécies Removidas: Na Versão 3 da lista, algumas espécies de plantas da Versão 2 foram excluídas devido à sua falta de importância como fontes alimentares.
(vi) Inclusão de Espécies: algumas espécies frutíferas listadas nos livros *Plantas para o Futuro* [8., 39.–41.], que estavam ausentes na Versão 2, foram incluídas na Versão 3.

### Banco de dados 2

O iNaturalist oferece ferramentas para gerenciar observações de interesse, conhecidas como Projetos [47.]. Utilizamos dois tipos de projetos:

- Projeto de Coleção: funciona como uma busca salva no banco de dados com recursos aprimorados para visualização de dados, como um banner distintivo, ícone, URL e um jornal que permite a comunicação com os seguidores do projeto [47.]. Ao criar um Projeto de Coleção, requisitos específicos podem ser definidos, como ”táxons, localizações, usuários, datas e grau de qualidade”. Toda vez que a página do projeto é acessada, ”o iNaturalist realiza uma busca rápida e exibe todas as observações que correspondem aos requisitos do projeto” [47.].
- Projeto Guarda-Chuva: são projetados para compilar, comparar ou promover vários projetos de coleção [47.].

Os requisitos para os projetos de coleção do *Pomar Urbano* foram definidos da seguinte maneira: a observação registrada pelo usuário deve ser sobre uma das espécies de plantas frutíferas na lista taxonômica compilada (veja a seção Disponibilidade de dados e materiais de apoio). Esta observação deve ocorrer em uma das capitais das 27 unidades federativas do Brasil. O projeto guarda-chuva foi configurado para acompanhar os dados de todos os 27 projetos de coleção [48.].

## Amostra do banco de dados

A Versão 3 do Banco de Dados 1 contém 429 espécies de plantas frutíferas. Notavelmente, 398 dessas espécies foram observadas por usuários do iNaturalist em várias localidades do Brasil até 22 de agosto de 2023. O *Pomar Urbano* acumulou uma coleção de 10.943 observações em todo o Brasil, referidas neste artigo como Banco de Dados 2. Este banco de dados foi gerado tanto a partir de registros oportunistas no iNaturalist, sem o uso de estratégias de engajamento ativo pelos coordenadores do projeto, quanto por meio da promoção de bioblitz.

A Figura 1 mostra as 10 cidades com o maior número de observações. Este ranking abrange todas as cinco macro-regiões do Brasil. Brasília (região Centro-Oeste) lidera esse ranking, seguida por São Paulo e Rio de Janeiro (ambas na região Sudeste).

**Figura 1.** Dez cidades brasileiras com o maior número de observações de espécies frutíferas. Nota: Captura de tela do projeto “Pomar Urbano” no iNaturalist, baseada em observações coletadas até agosto de 2023. Esta imagem representa os dados disponíveis naquele momento e pode não refletir as atualizações ou adições mais recentes. Para consultar os dados atualizados, visite a página do projeto em: https://www.inaturalist.org/projects/pomar-urbano.

 A Figura 2 mostra algumas observações de Brasília. Entre as dez espécies mais frequentemente observadas, sete são nativas do Brasil, nomeadamente *Caryocar brasiliense, Eugenia dysenterica, Psidium guajava, Eugenia uniflora, Bixa orellana, Hymenaea courbaril* e *Anacardium humile*. A espécie com o maior número de observações é *Caryocar brasiliense*, que é endêmica do Cerrado brasileiro, bioma que abrange todo o território de Brasília [49.]. Esta espécie em particular tem uma significativa importância cultural na culinária do Centro-Oeste brasileiro, segundo [50.], o que pode explicar sua ampla presença e disseminação por Brasília.

**Figura 2.** Dez espécies mais observadas em Brasília. Nota: Captura de tela do projeto “Pomar de Brasília” no iNaturalist, baseada em observações coletadas até agosto de 2023. Esta imagem representa os dados disponíveis naquele momento e pode não refletir as atualizações ou adições mais recentes. Para consultar os dados atualizados, visite a página do projeto em: https://www.inaturalist.org/projects/pomar-de-brasilia.

 A cidade de São Paulo apresenta um cenário distinto (veja a Figura 3). Entre as espécies mais observadas na cidade, apenas quatro delas (*Eugenia uniflora, Syagrus romanzoffiana, Plinia cauliflora* e *Psidium guajava*) são nativas do Brasil. Isso destaca uma diferença significativa na composição de espécies em comparação com Brasília.

Além disso, houve variações notáveis ao considerar as espécies que obtiveram o maior número de observações em cada cidade. Apenas *Psidium guajava, Eugenia uniflora* e *Persea americana* apareceram entre as espécies mais observadas tanto em Brasília quanto em São Paulo. Como essas cidades estão situadas em regiões caracterizadas por biomas distintos, diferenças na diversidade de espécies entre essas localidades eram esperadas. As características de cada bioma contribuem para a variação na composição e distribuição de espécies frutíferas, destacando a importância de considerar fatores ecológicos regionais ao analisar padrões de biodiversidade.

**Figura 3.** Dez espécies mais observadas em São Paulo. Nota 1: Captura de tela do projeto “Pomar Paulistano” no iNaturalist, baseada em observações coletadas até agosto de 2023. Esta imagem representa os dados disponíveis naquele momento e pode não refletir as atualizações ou adições mais recentes. Para consultar os dados atualizados, visite a página do projeto em: https://www.inaturalist.org/projects/pomar-paulistano. Nota 2: Conforme os avanços recentes na taxonomia, Plinia cauliflora foi sinonimizada com Plinia peruviana, nome este atualmente reconhecido como mais aceito, conforme documentado na Flora e Funga do Brasil [42.]. No entanto, até a data de publicação deste artigo, a atualização para Plinia peruviana ainda não havia sido refletida no iNaturalist, razão pela qual a figura apresenta o nome anterior, Plinia cauliflora.

 No Rio de Janeiro (veja a Figura 4), a abundância de espécies nativas de plantas frutíferas também é evidente. Entre as dez espécies mais observadas, sete delas são nativas do Brasil, incluindo *Eugenia uniflora, Syagrus romanzoffiana, Lecythis pisonis, Myrciaria glazioviana, Pachira aquatica, Opuntia monacantha* e *Eugenia brasiliensis*.

**Figura 4.** Dez espécies mais observadas no Rio de Janeiro. Nota: Captura de tela do projeto “Pomar Carioca” no iNaturalist, baseada em observações coletadas até agosto de 2023. Esta imagem representa os dados disponíveis naquele momento e pode não refletir as atualizações ou adições mais recentes. Para consultar os dados atualizados, visite a página do projeto em: https://www.inaturalist.org/projects/pomar-carioca.

 Tanto *Eugenia uniflora* quanto *Syagrus romanzoffiana* aparecem como espécies proeminentes entre as dez mais observadas nas três cidades. A prevalência de *Eugenia uniflora* pode ser atribuída à sua adaptabilidade a diferentes condições climáticas e de solo [51.]. Essa adaptabilidade, aliada ao crescente interesse econômico nas frutas produzidas por essa planta, levou ao seu cultivo em várias regiões do mundo, não apenas no Brasil [51.].

A significativa presença de *Syagrus romanzoffiana* nas três cidades pode ser atribuída à sua adaptabilidade aos biomas prevalentes nessas regiões, incluindo a Mata Atlântica e o Cerrado [52.]. A polpa e o amêndoa do fruto dessa espécie podem ser consumidos por humanos e também têm grande valor para diversos grupos de animais, indicando sua importância ecológica na provisão de recursos alimentares [52., 53.]. Além disso, comunidades nativas acreditam que essa espécie de palmeira é uma planta sagrada, atribuída com significado espiritual cosmogônico e cosmológico [53.]. Estudos recentes também revelaram o potencial medicinal de *Syagrus romanzoffiana*, demonstrando sua eficácia no tratamento de infecções e doenças crônicas [54.]. Adicionalmente, descobriu-se que as sementes desta planta possuem alta concentração de óleo, tornando-as uma candidata promissora para a produção de biodiesel [55.].

## Validação dos dados e controle de qualidade

A identificação de espécies no iNaturalist utiliza um Modelo de Visão Computacional (CVM) para processar imagens de observações e sugerir nomes potenciais de espécies [56.–58.]. Embora o CVM não possua dados sobre todas as 2 milhões de espécies registradas mundialmente, ele identifica com sucesso cerca de 85% das observações [57., 58.]. Para espécies não reconhecidas pelo CVM, ele recomenda classificações taxonômicas superiores, como gênero ou família [58.].

Um ponto forte do iNaturalist é sua comunidade de especialistas, conhecidos como identificadores, que aumentam significativamente a precisão da identificação de espécies na plataforma [56.]. Estes identificadores também contribuem para o CVM, fornecendo dados de qualidade e validando as identificações das observações [56.].

As contribuições contínuas desses identificadores são vitais para a melhoria contínua do CVM. Ao fornecerem um conjunto de dados de alta qualidade e expandirem a cobertura de espécies do modelo, eles aumentam significativamente seu desempenho geral. Para mais insights sobre o impacto dos identificadores na qualidade dos dados do iNaturalist, consulte [56.].

O iNaturalist aplica critérios de qualidade de dados, classificando os registros em vários níveis. O mais alto é o ”Grau de Pesquisa” (RG), caracterizado por critérios específicos, incluindo ter uma data válida, localização, foto ou gravação de som, ser de um organismo não cativo ou não cultivado e consenso da comunidade na identificação do organismo no nível de espécie ou inferior [59.]. O GBIF indexa observações RG, tornando-as acessíveis para download [60.].

No projeto *Pomar Urbano*, priorizar dados RG do iNaturalist seria lógico devido à necessidade de identificação precisa de espécies de plantas, especialmente quando há riscos à saúde decorrentes de frutas não comestíveis. No entanto, surge um desafio significativo, pois o projeto foca em plantas forrageáveis, muitas das quais são cultivadas e, portanto, não são elegíveis para o status RG no iNaturalist.

Esta categorização é particularmente complexa em ambientes urbanos, onde a linha entre plantas cultivadas e selvagens pode se confundir. Por exemplo, uma planta inicialmente cultivada por humanos pode se naturalizar em um ambiente urbano, levantando dúvidas sobre sua categorização. Essa ambiguidade é evidente nas discussões dentro da comunidade iNaturalist, onde há um debate contínuo sobre como lidar com observações de plantas que podem ter sido inicialmente plantadas, mas que se tornaram auto-sustentáveis e naturalizadas em um contexto urbano. Para mais informações sobre as complexidades em torno da classificação de plantas como “cultivadas” em contextos urbanos, consulte o fórum de discussão do iNaturalist [61.].

Dadas essas nuances, depender exclusivamente de dados RG no projeto *Pomar Urbano* poderia ser limitante. O foco do projeto em ambientes urbanos significa que muitas observações úteis podem ser de plantas cultivadas. Portanto, embora as observações RG sejam preferidas por sua precisão verificada, o projeto se beneficia de uma abordagem mais inclusiva, considerando também dados de observações não RG. Ainda assim, acreditamos que este conjunto de dados também poderia ser relevante para o GBIF e outras plataformas de dados de biodiversidade, considerando o potencial interesse em dados que esclarecem a distribuição e características de espécies de plantas economicamente importantes em ecossistemas urbanos.

## Potencial de reuso

Os conjuntos de dados apresentados neste artigo fornecem insumos para entender a diversidade de plantas frutíferas em cidades brasileiras selecionadas. Isso pode contribuir para várias questões de pesquisa abertas na literatura existente sobre forrageamento urbano e serviços ecossistêmicos em ambientes urbanos, incluindo, mas não se limitando a:

- Examinar como a composição atual de espécies nas cidades forma paisagens preexistentes que são comestíveis e úteis, como explorado nos trabalhos de [16., 17., 62.];
- Desenvolver fontes de dados abertos focadas em florestas alimentares urbanas, conforme destacado por [37.];
- Investigar o papel das florestas urbanas no apoio a serviços ecossistêmicos culturais, como discutido por [63.];
- Analisar o forrageamento urbano como meio de melhorar a segurança alimentar [64.–69.];
- Facilitar o compartilhamento de conhecimento ecológico, como mencionado em [70.];
- Contribuir para a resiliência socioecológica, como estudado por [71.];
- Auxiliar na reconexão de residentes urbanos com a natureza e biodiversidade, como descrito por [72.–74.].

No contexto do projeto Pomar Urbano, o reuso de dados inclui:

### Alimentação e nutrição

Para envolver e inspirar indivíduos a explorar o potencial culinário de frutas disponíveis localmente, firmamos parceria com o laboratório de alimentos Sustentarea da Universidade de São Paulo. Neste local, documentamos receitas que destacam as frutas mais frequentemente encontradas em São Paulo. Experimentos adicionais também foram realizados em Brasília. Estas receitas serão compartilhadas em plataformas de mídia social para incentivar as pessoas a prepararem pratos fáceis, acessíveis e saborosos usando esses ingredientes regionais. A Figura 5 mostra os resultados de alguns desses experimentos.

**Figura 5.** Receitas preparadas para o *Pomar Urbano*. (a) Salada verde com molho de maracujá (*Passiflora sp.*) e tortelli de taioba (*Xanthosoma taioba*) com molho de goiaba (*Psidium guajava*). Produção da receita: Ana Maria Bertolini, Gabriela Rigote, Natalie Marinho Dantas. Foto: Gabriela Rigote [48.]. (b) Geleia de jabuticaba (*Plinia peruviana*), preparada em dezembro de 2023 em Brasília. Preparação da receita e Foto: Lidio Coradin e Vera Coradin. (c) Mangas forrageadas, Brasília, dezembro de 2023. Esta fotografia mostra mangas maduras (*Mangifera indica*), colhidas à mão durante uma expedição de forrageamento. Foto: Lidio Coradin. (d) Chutney de manga preparado por Lidio Coradin e Vera Coradin com mangas coletadas em Brasília, dezembro de 2023. Foto: Lidio Coradin.

 À medida que nossa iniciativa avança, pretendemos expandir a coleção de receitas para incluir frutas de outras cidades. O livro *Biodiversidade Brasileira: sabores e aromas* [75.] também está sendo usado como referência para a elaboração de receitas brasileiras usando frutas.

### Indústria criativa

A indústria criativa, também conhecida como economia criativa, abrange uma diversidade de setores dedicados a gerar e comercializar criatividade, arte e conteúdo original. Esta indústria inclui publicidade, arquitetura, artes e ofícios, design (incluindo moda), cinema, música, artes cênicas, publicações e software (como videogames) [76.]. Ela prospera na inovação e atende às demandas culturais e econômicas, muitas vezes desfocando as linhas entre arte e comércio. A indústria criativa pode se beneficiar dos dados apresentados neste artigo de diversas maneiras.

Atualmente, está sendo realizada a identificação de influenciadores digitais brasileiros em plataformas de destaque, tais como TikTok e Instagram, com foco naqueles que discutem temas relacionados a dietas sustentáveis, vegetarianismo, ciência e natureza. O propósito desta ação é estabelecer uma colaboração com esses influenciadores, visando obter seu apoio para divulgar nossa iniciativa junto aos seus seguidores. Utilizando os dados provenientes de nosso projeto, tais influenciadores podem criar conteúdos relevantes, enquanto simultaneamente contribuem para a ampliação da participação dos usuários em nossas atividades de coleta de dados.

Além da criação de conteúdo, informações sobre plantas frutíferas podem inspirar o desenvolvimento de diversos produtos. A Figura 6 exibe produtos criados por artistas brasileiros inspirados em observações registradas no *Pomar Urbano*.

**Figura 6.** Designs de produtos inspirados no *Pomar Urbano*. (a) Estampa localizada em uma caneca de porcelana inspirada na flor do cacto da noite (*Epiphyllum oxypetalum*), descoberta durante expedição na cidade de São Paulo. Criada por um grupo de estudantes de Design de Moda do Centro Universitário Belas Artes de São Paulo, sob a supervisão de Maria Carolina Garcia [48.]. (b) Estampa em toalha de mesa inspirada na flor da goiabeira (*Psidium guajava*), usando a técnica tradicional da Chita brasileira. Criada por Luciana Mendonça, estudante de Design de Interiores do Centro Universitário Belas Artes de São Paulo sob a supervisão de Maria Carolina Garcia [48.]. (c) Ilustrações digitais exibindo diversas plantas brasileiras (do canto superior esquerdo para o canto inferior direito: *Annona mucosa, Mauritia flexuosa, Campomanesia phaea, Theobroma grandiflorum, Campomanesia xanthocarpa* e *Plinia peruviana*). Projetadas para o projeto Pomar Urbano com base em várias fotos obtidas do iNaturalist.org. Ilustrações: Bruna Stefani Perin e Fernanda Beatriz Fernandez Correa. Supervisão: Rubens Rangel Silva (Ânima Educação).

### Compreendendo a fenologia das plantas

Numerosas fontes de referência fornecem informações sobre o período de frutificação e floração de várias espécies no Brasil. O trabalho realizado por Coradin, Camillo e Vieira [8.] é notável, apresentando uma extensa tabela que detalha os aspectos da fenologia das plantas mencionadas, incluindo seus picos, para espécies encontradas no Norte do Brasil. A partir desses recursos literários existentes, antecipamos a utilização dos dados fornecidos para apoiar nossas futuras análises sobre as estações de frutificação de cada espécie.

Nosso principal objetivo é comparar os dados coletados por cientistas cidadãos com as descobertas apresentadas por fontes de referência. Por meio desta análise comparativa, pretendemos verificar se as informações coletadas por meio de iniciativas de CS produzem resultados semelhantes aos alcançados por cientistas profissionais. Esta investigação visa avaliar a precisão e confiabilidade da coleta de dados da CS dentro do contexto específico do Pomar Urbano.

Ao examinar e contrastar sistematicamente esses resultados, podemos elucidar a eficácia e o potencial da CS como meio de coleta de dados para tais projetos. Esta comparação servirá como um passo crucial para aprimorar nosso entendimento dos padrões sazonais de frutos, ao mesmo tempo em que destaca as contribuições que os cientistas cidadãos podem fazer no avanço do conhecimento científico. Além disso, esses dados podem potencialmente destacar mudanças temporais na sazonalidade reprodutiva das espécies de plantas monitoradas a longo prazo.

### Aplicações de aprendizado de máquina

A segurança e a autenticidade dos alimentos podem ser asseguradas por meio de dados derivados de plantas frutíferas, servindo como uma referência fundamental para os atributos genuínos das frutas. Algoritmos de Aprendizado de Máquina (ML) podem então ser empregados para identificar irregularidades na qualidade ou composição das frutas, permitindo a detecção de práticas enganosas e potenciais riscos à segurança alimentar [77.]. Além disso, em ambientes urbanos, a produção local de frutas e vegetais pode ser integrada de maneira eficaz à cadeia de suprimentos urbana, aprimorando a gestão da cadeia de suprimentos. Algoritmos de ML podem otimizar a logística de transporte e minimizar o desperdício de alimentos, levando a um sistema alimentar urbano mais sustentável e eficiente. Ademais, diante de desafios ambientais, o ML oferece uma ferramenta poderosa para analisar dados de longo prazo de culturas de frutas urbanas. Essa abordagem baseada em dados pode fornecer informações valiosas para entender o impacto das mudanças climáticas e da urbanização no crescimento das plantas e na biodiversidade. Tal conhecimento pode ser crucial para orientar decisões de planejamento urbano e iniciativas de conservação.

Em conclusão, quando combinada com as capacidades de ML, a riqueza de dados produzidos sobre plantas frutíferas em ambientes urbanos pode ter um impacto significativo em questões relacionadas à autenticidade, segurança dos alimentos e preocupações ambientais. Essas aplicações aprimoram a qualidade de vida urbana e contribuem para os esforços globais de construir ecossistemas urbanos sustentáveis e resilientes.

Para insights adicionais sobre ciência cidadã para a coleta de alimentos em meio urbano, consulte o artigo relacionado na revista GigaScience [78.].

## Data Availability

Os bancos de dados estão disponíveis no Zenodo: Banco de Dados [1.] e Banco de Dados [2.]. Os dados também estão disponíveis através do repositório GigaDB [79.].

## Lista de abreviações

IA: Inteligência Artificial; CS: Ciência Cidadã; MCV: Modelo de Visão Computacional; RG: Grau de Pesquisa.

## Declarações

### Contribuições dos autores

Conceitualização do Estudo: FMS, MCG, AMS, LFP, BCMSM. Curadoria de Dados: FMS, LC, MCPC, ACSL, SGMF, HDEG, BJS. Análise Formal: FMS, RRS, LFP, NPGL, LC. Aquisição de Financiamento: AMS, UBM, BCMSM, MCG. Investigação: FMS, LFP, MCG, LC, NPGL, RRS, AMC, BCMSM, SK, UBM, DPD, RS, AMS. Metodologia: FMS, NPGL, SK, UBM. Administração do Projeto: FMS, AMS, MCG. Supervisão: AMS, LFP, BCMSM, UBM, AMC, MCG. Validação: LC. Visualização de Dados: FMS, RRS. Escrita – rascunho original: FMS, LFP, LC, NPGL, AG, YB, SK. Escrita – revisão e edição: Todos os autores contribuíram significativamente para a revisão e edição deste manuscrito.

### Financiamento

FMS agradece à Fundação de Amparo à Pesquisa do Estado de São Paulo (FAPESP) (Número do processo: 21/15125-0, e 22/08385-8). BCMSM agradece ao Conselho Nacional de Desenvolvimento Científico e Tecnológico (CNPq) (Número do processo: 303650/2019-2). AMS, NPGL, SK e FMS agradecem à FAPESP (Número do processo: 2018/14994-1). SK agradece à FAPESP (Número do processo: 2019/26760-8).

## References

[1] SoaresFM, CoradinL, LimaACS Taxonomic list of Brazilian fruit-bearing plants for human use (Version 3.0). Zenodo, November 2023; 10.5281/zenodo.10212850.

[2] SoaresFM, PiresLF, GarciaMC Fruit-bearing plant species observations in Brazilian cities (Version 2.0). Zenodo, August 2023; 10.5281/zenodo.8273049.

[3] WieczorekJ, BloomD, GuralnickR Darwin core: an evolving community-developed biodiversity data standard. PloS One, 2012; 7(1): e29715. doi:10.1371/journal.pone.0029715.22238640 PMC3253084

[4] YessonC, BrewerPW, SuttonT How global is the global biodiversity information facility? PloS One, 2007; 2(11): e1124. doi:10.1371/journal.pone.0001124.17987112 PMC2043490

[5] IvanovaNV, ShashkovMP. The possibilities of GBIF data use in ecological research. Russ. J. Ecol., 2021; 52(1): 1–8. doi:10.1134/S1067413621010069.

[6] LaneMA, EdwardsJL. The global biodiversity information facility (GBIF). In: CurryGB, HumphriesCJ (eds), Biodiversity Databases: Techniques, Politics, and Applications. Boca Raton, London, New York: CRC Press, 2007. doi:10.1201/9781439832547.

[7] United Nations Environment Programme. Megadiverse Brazil giving biodiversity an online boost. https://www.unep.org/news-and-stories/story/megadiverse-brazil-giving-biodiversity-online-boost#:∼:text=Brazil%5C%20is%5C%20at%5C%20the%5C%20top,more%5C%20than%5C%204%5C%2C000%5C%20plant%5C%20species (visited on 05/26/2023).

[8] CoradinL, CamilloJ, VieiraICG (eds) Especies nativas da flora brasileira de valor econômico atual ou potencial: plantas para o futuro – Regiao Norte. Ministério do Meio Ambiente, 2022.

[9] GomesSM, ChavesVM, de CarvalhoAM Biodiversity is overlooked in the diets of different social groups in brazil. Sci. Rep., 2023; 13(1): 7509. doi:10.1038/s41598-023-34543-8.37160999 PMC10170146

[10] ClarkKH, NicholasKA. Introducing urban food forestry: a multifunctional approach to increase food security and provide ecosystem services. Landsc. Ecol., 2013; 28(9): 1649–1669. doi:10.1007/s10980-013-9903-z.

[11] Food and Agriculture Organization of the United Nations. Unasylva 250: Forests and Sustainable Cities. FAO Unasylva, 2018. https://www.fao.org/documents/card/es/c/I8707EN.

[12] SalbitanoF, BorelliS, ConigliaroM Guidelines on urban and peri-urban forestry. In: FAO Forestry Paper. vol. 178, Food and Agriculture Organization of the United Nations (FAO), 2016. https://www.fao.org/documents/card/es/c/e068e0d9-0c97-41c7-a856-05556a1bd10b.

[13] LwasaS, MugaggaF, WahabB Urban and peri-urban agriculture and forestry: Transcending poverty alleviation to climate change mitigation and adaptation. Urban Clim., 2014; 7: 92–106. doi:10.1016/j.uclim.2013.10.007.

[14] BatistaDAM, MacielEA, MachadoDL Urban greening dynamics in a brazil central city as a subsidy for public policies. Urban Ecosyst., 2023; 26: 845–856. doi:10.1007/s11252-023-01344-w.

[15] FreitasSR, TambosiLR, Ghilardi-LopesNP Spatial and temporal variation of potential resource availability provided by street trees in southeastern Brazil. Urban Ecosyst., 2020; 23(5): 1051–1062. doi:10.1007/s11252-020-00974-8.

[16] HurleyPT, EmeryMR, McLainR Whose urban forest? The political ecology of foraging urban nontimber forest products. In: IsenhourC, McDonoghG, CheckerM (eds), Sustainability in the Global City. 1st ed, Cambridge University Press, 2015; pp. 187–212, doi:10.1017/CBO9781139923316.014.

[17] HurleyPT, EmeryMR. Locating provisioning ecosystem services in urban forests: Forageable woody species in New York City, USA. Landsc. Urban Plan., 2018; 170: 266–275. doi:10.1016/j.landurbplan.2017.09.025.

[18] iNaturalist. [inaturalist]: What is it. https://www.inaturalist.org/pages/what+is+it (visited on 06/05/2023).

[19] KhapuginAA, KuzminIV, IvanovaLA. Distribution of four alien plants in Tyumen Region (Western Siberia): contribution of citizen science and expert data. Wulfenia, 2021; 28: 151–160. doi:10.5281/zenodo.6326883.

[20] BalashovI, MarkovaA. Expansion of the invasive balkan slug *Tandonia Kusceri* (stylommatophora: Milacidae): A new frontier in northern ukraine and other new records. Folia Malacolo., 2023; 31(1): 24–31. doi:10.12657/folmal.031.004.

[21] PrudicKL, OliverJC, BrownBV Comparisons of citizen science data-gathering approaches to evaluate urban butterfly diversity. Insects, 2018; 9(4): 186. doi:10.3390/insects9040186.30563191 PMC6316785

[22] MapeN, AlistoL, KitchingI. Hawkmoths of baguio city, philippines: a preliminary checklist derived from photo-based observation records available on iNaturalist and philippine lepidoptera internet platforms. Philipp. J. Sci., 2022; 151(4): 1447–1453. doi:10.56899/151.04.12.

[23] FogelNS, ThompsonMF, MunizPA The prevalence and manifestation of wing de-melanization in the eastern carpenter bee (Hymenoptera, Apidae Xylocopa virginica) associated with urban areas. J. Kans. Entomol. Soc., 2023; 94(4): 235–266. doi:10.2317/0022-8567-94.4.235.

[24] VardiR, Berger-TalO, RollU. iNaturalist insights illuminate COVID-19 effects on large mammals in urban centers. Biol. Conserv., 2021; 254: 108953. doi:10.1016/j.biocon.2021.108953.33424027 PMC7784545

[25] MuellerMA, DrakeD, AllenML. Using citizen science to inform urban canid management. Landsc. Urban Plan., 2019; 189: 362–371. doi:10.1016/j.landurbplan.2019.04.023.

[26] DrakeD, DubayS, AllenML. Evaluating human–coyote encounters in an urban landscape using citizen science. J. Urban Ecol., 2021; 7(1): juaa032. doi:10.1093/jue/juaa032.

[27] LopezB, MinorE, CrooksA. Insights into human-wildlife interactions in cities from bird sightings recorded online. Landsc. Urban Plan., 2020; 196: 103742. doi:10.1016/j.landurbplan.2019.103742.

[28] CerveraPEN. Anfibios Y reptiles en parques recreativos ecológicos de la ciudad de mérida, yucatán, méxico. Revista Latinoamericana de Herpetología, 2021; 4(1): 82–94. doi:10.22201/fc.25942158e.2021.1.196.

[29] JonesD, FosheeB, FitzgeraldL. A herpetological survey of edith l. Moore nature sanctuary. Check List, 2021; 17(1): 27–38. doi:10.15560/17.1.27.

[30] HerreraDJ, CoveMV. Camera trap serendipity and citizen science point to broader effects of urban heat islands on food webs. Food Webs, 2020; 25: e00176. doi:10.1016/j.fooweb.2020.e00176.

[31] Marín-GómezOH, Rodríguez FloresC, del Coro ArizmendiM. Assessing ecological interactions in urban areas using citizen science data: Insights from hummingbird–plant meta-networks in a tropical megacity. Urban For. Urban Green., 2022; 74: 127658. doi:10.1016/j.ufug.2022.127658.

[32] PutmanBJ, WilliamsR, LiE The power of community science to quantify ecological interactions in cities. Sci. Rep., 2021; 11(1): 3069. doi:10.1038/s41598-021-82491-y.33542360 PMC7862361

[33] CallaghanCT, OzeroffI, HitchcockC Capitalizing on opportunistic citizen science data to monitor urban biodiversity: A multi-taxa framework. Biol. Conserv., 2020; 251: 108753. doi:10.1016/j.biocon.2020.108753.

[34] LiE, ParkerSS, PaulyGB An urban biodiversity assessment framework that combines an urban habitat classification scheme and citizen science data. Front. Ecol. Evol., 2019; 7: 277. doi:10.3389/fevo.2019.00277.

[35] CambriaVE, CampagnaroT, TrentanoviG Citizen science data to measure human use of green areas and forests in european cities. Forests, 2021; 12(6): 779. doi:10.3390/f12060779.

[36] PrudicKL, WilsonJK, ToshackMC Creating the urban farmer’s almanac with citizen science data. Insects, 2019; 10(9): 294. doi:10.3390/insects10090294.31514459 PMC6780957

[37] StarkPB, MillerD, CarlsonTJ Open-source food: nutrition, toxicology, and availability of wild edible greens in the east bay. PloS One, 2019; 14(1): e0202450. doi:10.1371/journal.pone.0202450.30653545 PMC6336281

[38] LorenziH, BacherL, LacerdaM Frutas brasileiras e exóticas cultivadas. Instituto Plantarum, 2006.

[39] CoradinL, SiminskiA, ReisA (eds) Espécies nativas da flora brasileira de valor econômico atual ou potencial: plantas para o futuro – Região Sul. Ministério do Meio Ambiente, 2011.

[40] VieiraRF, CamilloJ, CoradinL (eds) Espécies nativas da flora brasileira de valor econômico atual ou potencial: plantas para o futuro – Região Centro-Oeste. Ministério do Meio Ambiente, 2016.

[41] CoradinL, CamilloJ, PareynFGC (eds) Espécies nativas da flora brasileira de valor econômico atual ou potencial: plantas para o futuro – Região Nordeste. Ministério do Meio Ambiente, 2018.

[42] do Rio de JaneiroJB. Flora e funga do brasil. 2024; http://floradobrasil.jbrj.gov.br/ (Accessed 31 January 2024).

[43] Missouri Botanical Garden. Tropicos.org. 2024; https://tropicos.org (Accessed 31 January 2024).

[44] The Royal Botanic Gardens, Kew and Harvard University Herbaria & Libraries and Australian National Herbarium. International plant names index. 2023; http://www.ipni.org (Accessed 25 July 2023).

[45] Royal Botanic Gardens, Kew. Plants of the world online. 2024; http://www.plantsoftheworldonline.org/ (Accessed 31 January 2024).

[46] New York Botanical Garden. Vascular plant types catalog. 2024; https://sciweb.nybg.org/science2/hcol/vasc/index.asp.html (Accessed 31 January 2024).

[47] iNaturalist. Managing projects. https://www.inaturalist.org/pages/managing-projects (visited on 06/05/2023).

[48] SoaresFM, PiresLF, GarciaMC Optimizing the monitoring of urban fruit-bearing flora with citizen science: An overview of the pomar urbano initiative. Biodivers. Inf. Sci. Stand., 2023; 7: e112009. doi:10.3897/biss.7.112009.

[49] Governo do Distrito Federal. Geografia. https://www.df.gov.br/333/ (visited on 05/27/2023).

[50] AntunesMJM, dos SantosVVO, de PaulaVA. Culinária do Centro-Oeste: o pequi como iguaria natural. Etec Profa. Anna de Oliveira Ferraz, 2020. http://ric.cps.sp.gov.br/handle/123456789/7160.

[51] BezerraJEF, de Lira JuniorJS, da Silva JuniorJF. *Eugenia uniflora*: pitanga. In: CoradinL, CamilloJ, PareynFGC (eds), Espécies nativas da flora brasileira de valor econômico atual ou potencial: plantas para o futuro: região Nordeste. Ministério do Meio Ambiente, 2018; pp. 155–169.

[52] CarvalhoPER. Jerivá: *Syagrus romanzoffiana* . In: Espécies Arbóreas Brasileiras. Embrapa, 2006; pp. 309–315.

[53] JorgeN, da SilvaAC, VeroneziCM. Syagrus romanzoffiana. In: de LimaFF, LescanoCH, de OliveiraIP (eds), Fruits of the Brazilian Cerrado: Composition and Functional Benefits. Springer International Publishing, 2021; pp. 141–159, doi:10.1007/978-3-030-62949-6_9.

[54] de Lacerda CoriolanoD, AlvesMHME, CavalcantiIMF. Biological potential of products obtained from palm trees of the genus syagrus. Evid. Based Complementary Altern. Med., 2021; 2021: 1–11. doi:10.1155/2021/5580126.PMC839756434457025

[55] MoreiraMAC, Payret ArrúaME, AntunesAC Characterization of *Syagrus romanzoffiana* oil aiming at biodiesel production. Ind. Crops Prod., 2013; 48: 57–60. doi:10.1016/j.indcrop.2013.04.006.

[56] CampbellCJ, BarveV, BelitzMW Identifying the identifiers: how inaturalist facilitates collaborative, research-relevant data generation and why it matters for biodiversity science. BioScience, 2023; 73(7): 533–541. doi:10.1093/biosci/biad051.

[57] UedaK-I. An overview of computer vision in iNaturalist. Biodivers. Inf. Sci. Stand., 2020; 4: e59133. doi:10.3897/biss.4.59133.

[58] iNaturalist. iNaturalist computer vision explorations. 2022; https://www.inaturalist.org/pages/computer_vision_demo (visited on 01/02/2023).

[59] iNaturalist. What is the data quality assessment and how do observations qualify to become “research grade”? https://www.inaturalist.org/pages/help#quality (visited on 05/27/2023).

[60] iNaturalist Contributors. iNaturalist research-grade observations. 2023; 10.15468/AB3S5X.

[61] SponslerD. The category of “cultivated” is problematic for plants in urban landscapes. *iNatForum*. April 2019; https://forum.inaturalist.org/t/the-category-of-cultivated-is-problematic-for-plants-in-urban-landscapes/2317 (Accessed 31 January 2024).

[62] HurleyPT, BeckerS, EmeryMR Estimating the alignment of tree species composition with foraging practice in philadelphia’s urban forest: Toward a rapid assessment of provisioning services. Urban For. Urban Green., 2022; 68: 127456. doi:10.1016/j.ufug.2021.127456.

[63] PlieningerT, BielingC, FagerholmN The role of cultural ecosystem services in landscape management and planning. Curr. Opin. Environ. Sustain., 2015; 14: 28–33. doi:10.1016/j.cosust.2015.02.006.

[64] SynkCM, KimBF, DavisCA Gathering baltimore’s bounty: characterizing behaviors, motivations, and barriers of foragers in an urban ecosystem. Urban For. Urban Green., 2017; 28: 97–102. doi:10.1016/j.ufug.2017.10.007.

[65] BungeA, DiemontSAW, BungeJA Urban foraging for food security and sovereignty: quantifying edible forest yield in syracuse, new york using four common fruit- and nut-producing street tree species. J. Urban Ecol., 2019; 5(1): juy028. doi:10.1093/jue/juy028.

[66] GarekaeH, ShackletonCM. Urban foraging of wild plants in two medium-sized south african towns: People, perceptions and practices. Urban For. Urban Green., 2020; 49: 126581. doi:10.1016/j.ufug.2020.126581.

[67] SardeshpandeM, ShackletonC. Fruits of the city: the nature, nurture and future of urban foraging. People and Nat., 2023; 5(1): 213–227. doi:10.1002/pan3.10428.

[68] BritoVV, BorelliS. Urban food forestry and its role to increase food security: A brazilian overview and its potentialities. Urban For. Urban Green., 2020; 56: 126835. doi:10.1016/j.ufug.2020.126835.

[69] GarekaeH, ShackletonCM. Foraging wild food in urban spaces: the contribution of wild foods to urban dietary diversity in south africa. Sustainability, 2020; 12(2): 678. doi:10.3390/su12020678.

[70] Landor-YamagataJ, KowarikI, FischerL. Urban foraging in berlin: people, plants and practices within the metropolitan green infrastructure. Sustainability, 2018; 10(6): 1873. doi:10.3390/su10061873.

[71] SardeshpandeM, HurleyPT, MolleeE How people foraging in urban greenspace can mobilize social–ecological resilience during covid-19 and beyond. Front. Sustain. Cities, 2021; 3: 686254. doi:10.3389/frsc.2021.686254.

[72] PalliwodaJ, KowarikI, Von Der LippeM. Human-biodiversity interactions in urban parks: The species level matters. Landsc. Urban Plan., 2017; 157: 394–406. doi:10.1016/j.landurbplan.2016.09.003.

[73] FischerLK, KowarikI. Connecting people to biodiversity in cities of tomorrow: Is urban foraging a powerful tool? Ecol. Indic., 2020; 112: 106087. doi:10.1016/j.ecolind.2020.106087.

[74] SchunkoC, BrandnerA. Urban nature at the fingertips: investigating wild food foraging to enable nature interactions of urban dwellers. Ambio, 2022; 51(5): 1168–1178. doi:10.1007/s13280-021-01648-1.34709589 PMC8552430

[75] SantiagoRdAC, CoradinL (eds) Biodiversidade brasileira: sabores e aromas. Ministério do Meio Ambiente, 2018.

[76] Urban 20. Championing ‘Local’ Culture and Economy. Urban 20 India, 2023. https://www.u20india.org/Content/WhitePaper/6_CLCE_White%20Paper_1.pdf (Accessed 15 January 2024).

[77] MarvinHJP, HoenderdaalW, GavaiAK Global media as an early warning tool for food fraud; an assessment of MedISys-FF. Food Control, 2022; 137: 108961. doi:10.1016/j.foodcont.2022.108961.

[78] SoaresFM, PiresLF, GarciaMC Leveraging citizen science for monitoring urban forageable plants. GigaScience, 2024; doi:10.1093/gigascience/giae007.PMC1091421538442146

[79] SoaresFM, PiresLF, GarciaMC Supporting data for “Citizen science data on urban forageable plants: a case study in Brazil”. GigaScience Database, 2024; 10.5524/102499.
